# Proteomic analysis of plasma at the preterminal stage of rhesus nonhuman primates exposed to a lethal total-body dose of gamma-radiation

**DOI:** 10.1038/s41598-024-64316-w

**Published:** 2024-06-12

**Authors:** Alana D. Carpenter, Oluseyi O. Fatanmi, Stephen Y. Wise, John B. Tyburski, Amrita K. Cheema, Vijay K. Singh

**Affiliations:** 1 Division of Radioprotectants, Department of Pharmacology and Molecular Therapeutics, F. Edward Hébert School of Medicine “America’s Medical School”, Uniformed Services University of the Health Sciences, 4301 Jones Bridge Road, Bethesda, MD USA; 2 Armed Forces Radiobiology Research Institute, Uniformed Services University of the Health Sciences, Bethesda, MD USA; 3 Nelson Scientific Labs, LLC, Potomac, MD USA; 4 Department of Oncology, Lombardi Comprehensive Cancer Center, Georgetown University Medical Center, Washington, DC USA; 5 Department of Biochemistry, Molecular and Cellular Biology, Georgetown University Medical Center, Washington, DC USA

**Keywords:** Biomarkers, Gamma-radiation, Nonhuman primates, Preterminal, Proteomics, Total-body irradiation, Prognostic markers, Proteomics

## Abstract

The identification and validation of radiation biomarkers is critical for assessing the radiation dose received in exposed individuals and for developing radiation medical countermeasures that can be used to treat acute radiation syndrome (ARS). Additionally, a fundamental understanding of the effects of radiation injury could further aid in the identification and development of therapeutic targets for mitigating radiation damage. In this study, blood samples were collected from fourteen male nonhuman primates (NHPs) that were exposed to 7.2 Gy ionizing radiation at various time points (seven days prior to irradiation; 1, 13, and 25 days post-irradiation; and immediately prior to the euthanasia of moribund (preterminal) animals). Plasma was isolated from these samples and was analyzed using a liquid chromatography tandem mass spectrometry approach in an effort to determine the effects of radiation on plasma proteomic profiles. The primary objective was to determine if the radiation-induced expression of specific proteins could serve as an early predictor for health decline leading to a preterminal phenotype. Our results suggest that radiation induced a complex temporal response in which some features exhibit upregulation while others trend downward. These statistically significantly altered features varied from pre-irradiation levels by as much as tenfold. Specifically, we found the expression of integrin alpha and thrombospondin correlated in peripheral blood with the preterminal stage. The differential expression of these proteins implicates dysregulation of biological processes such as hemostasis, inflammation, and immune response that could be leveraged for mitigating radiation-induced adverse effects.

## Introduction

Potential exposure to high doses of ionizing radiation is an ever-increasing risk that is compounded by a global push toward clean, nuclear energy as well as by strained international relations between developed countries^1^. Nuclear events are not only detrimental in terms of the catastrophic damage they cause to infrastructure but are also particularly complicated to manage in terms of health, both in short- and long-terms^2,3^. Exposure to ionizing radiation damages living tissue directly by inducing double-strand breaks in DNA or indirectly by producing free radicals and reactive oxygen species (ROS)^4^. Direct damage to DNA is particularly detrimental as it alters gene expression, which induces a cascade of changes that can be observed downstream in the form of proteomic changes. Currently, there is no way for radiation doses to be accurately assessed in acutely exposed individuals that go on to develop acute radiation syndrome (ARS). Therefore, it is extremely difficult to treat and manage this illness^5^.

ARS is challenging to treat, not only due to difficulty in assessing absorbed radiation doses but also due to a prodromal and latent stage that lasts several days or even a few weeks before symptoms manifest^6,7^. In an attempt to address these shortcomings in patient care, research has been conducted to identify biomarkers (metabolites, proteins, etc.) in easily attainable samples (plasma or serum from blood samples) that can possibly assist in pinpointing the absorbed radiation dose in exposed individuals or anticipating health decline so appropriate treatments can be administered^8,9^. Biomarkers have been at the forefront of discussion and research within the radiation biology community for several years, as they can potentially exhibit biological processes closely related to the mechanism of disease^4,10,11^. In radiation exposed individuals, biomarkers that can be used to assess absorbed radiation doses as well as predict health decline are needed so that treatments can be applied to ultimately improve overall patient outcome^12–14^.

Biomarkers can be used in the diagnostic, prognostic, predictive, and pharmacodynamic aspects of drug development. One biomarker may play a role in more than one aspect of drug development. A *diagnostic* biomarker is a disease characteristic that categorizes an individual by the presence or absence of a physiological or pathophysiological state. A *prognostic* biomarker is a baseline attribute that categorizes victims by degree of risk for disease occurrence or progression of a disease. It is informative about the natural history of the disease in the absence of a therapeutic intervention. A *predictive* biomarker is a baseline characteristic that categorizes individuals by their likelihood of response to a particular treatment. A change in a *pharmacodynamic* biomarker indicates that a biological response has occurred in an individual who has received a drug; the magnitude of the change is considered pertinent to the response. From a regulatory viewpoint, biomarkers have been accepted through several ad hoc pathways in drug regulatory agencies. At the United States Food and Drug Administration (US FDA), the European Medicines Agency (EMEA) and the Pharmaceuticals and Medical Devices Agency (PMDA, Japan), biomarkers have been qualified in recent years. Currently, several biomarkers are approved for specific individual injuries; the US FDA has biomarkers for about 150 drug interactions validated, the EMEA has biomarkers for a few injuries approved, and the PMDA also has biomarkers for a few injuries accepted^15–17^. However, none of these are biomarkers for radiation injury. Multiple potential biomarkers are in the process of being confirmed, including some with radiation applications^18–20^.

**Figure 1:**
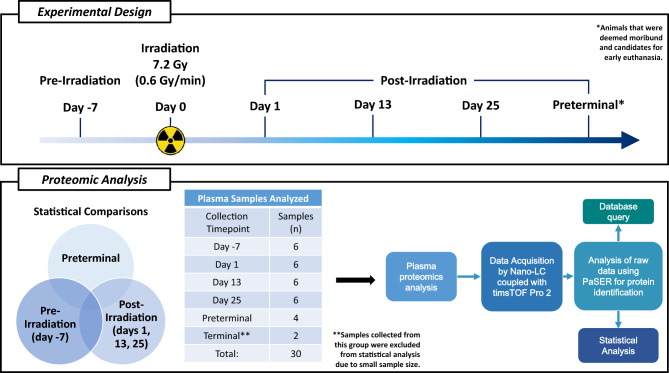
Experimental design to assess changes in plasma proteomics profiles in NHPs exposed to 7.2 Gy total-body irradiation (TBI) from samples collected pre-irradiation (day -7), post-irradiation (day 1, day 13, and day 25), or immediately prior to euthanasia (preterminal).

The current study attempts to elucidate the proteomic and biochemical landscape modulations in the blood plasma of nonhuman primates (NHPs) after exposure to a lethal dose of 7.2 Gy total-body radiation (Fig. 1). Plasma samples were collected pre-irradiation (day -7), at 1, 13, and 25 days post-irradiation, and immediately prior to death in a subgroup of moribund animals (termed “preterminal” samples)^21^. The comparative analysis of the plasma proteomic profiles at various time points was central to this investigation, as it provides insight into the temporal dynamics of radiation-induced biological alterations. Previous transcriptomic and metabolomic research has determined that there are definitive proteomic signatures in preterminal statuses, which warrant additional investigations^21,22^. Our results demonstrate dynamic changes in proteomic expression that evolved from acute to late post-irradiation phases. Interestingly, the preterminal phase was marked by an amplification of specific proteomic changes, indicating heightened biological stress or damage responses, particularly in proteins related to inflammation, hemostasis, and cellular integrity. The findings from this study contribute to a deeper understanding of the temporal progression of radiation injury and may aid in the identification of therapeutic targets for mitigating radiation damage.

## Results

**Figure 2:**
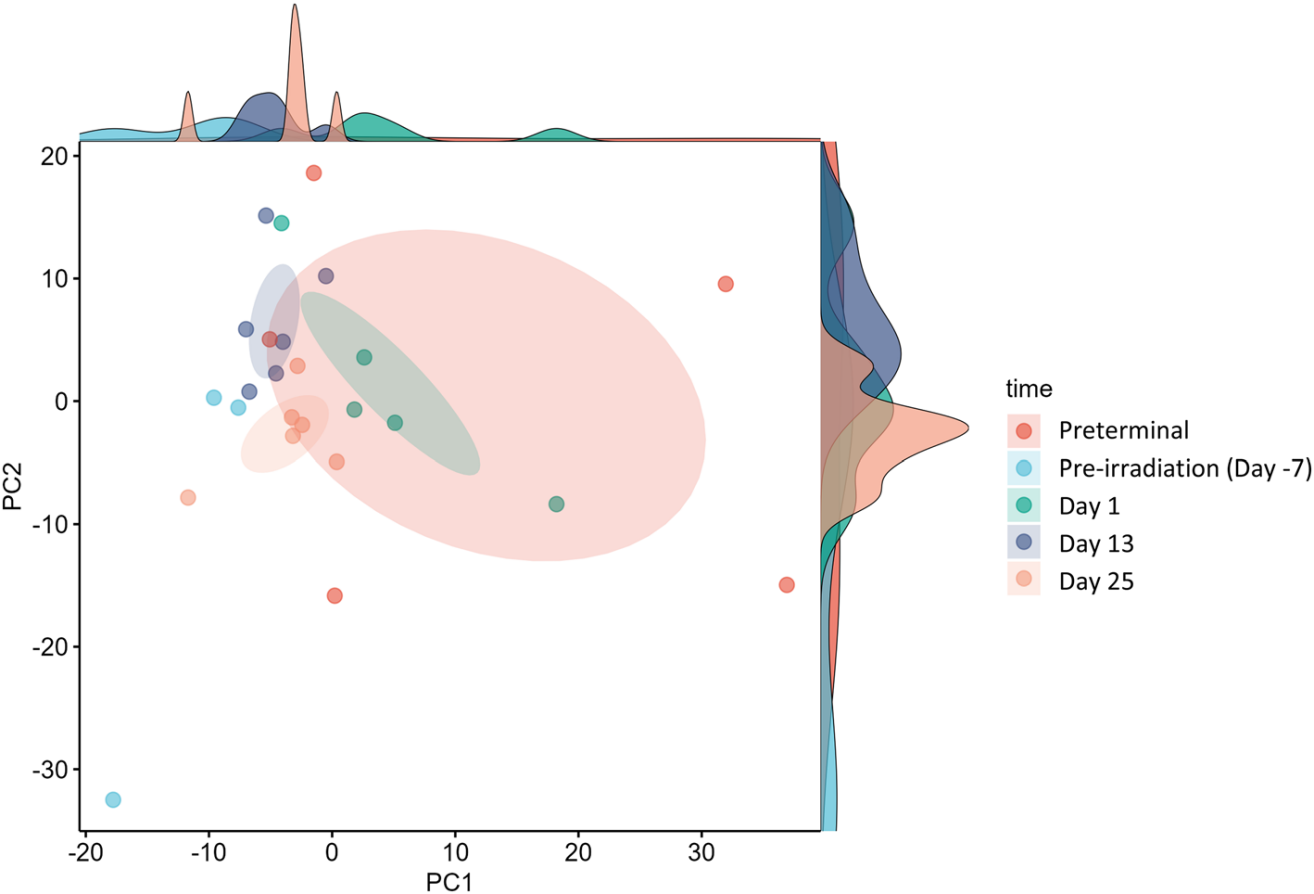
Principal Component Analysis (PCA) plot demonstrating the effect of 7.2 Gy TBI on plasma detected features. As is seen in the figure, preterminal scores are categorized distinctly from the rest of the samples in the dataset suggesting robust alterations in proteomic profiles.

For this study, we aimed to discern proteomic changes induced by radiation exposure by comparing samples collected post-irradiation to the pre-irradiation time point. Additionally, we wanted to determine whether there were significant changes in the proteomes of samples collected immediately prior to the euthanasia of moribund animals when compared to pre-irradiation and post-irradiation time points. Plasma samples were collected from 14 male NHPs pre-irradiation (day -7; n = 6), post-irradiation (days 1, 13, and 25; n = 6 for each time point), and immediately prior to humane euthanasia (preterminal; n = 4). To minimize the likelihood of false positives, multiple testing correction was performed by applying the False Discovery Rate (FDR) method to adjust p-values. Following the analysis and examination of plasma profiles, a stark proteomic contrast was observed between the pre-irradiation time point and the post-irradiation time points. This divergence between time points signifies a clear impact of radiation on the plasma proteome, as reflected in the principal component analysis (PCA) plot by the separate clusters formed by the pre-irradiation group compared to the day 1, day 13, day 25, and preterminal groups (Fig. 2). The effects of radiation on proteomic profiles are discussed separately from the preterminal samples, as we aimed to determine the presence of definitive preterminal proteomic signatures, if any.

### Radiation induced changes in proteomic profiles

**Figure 3:**
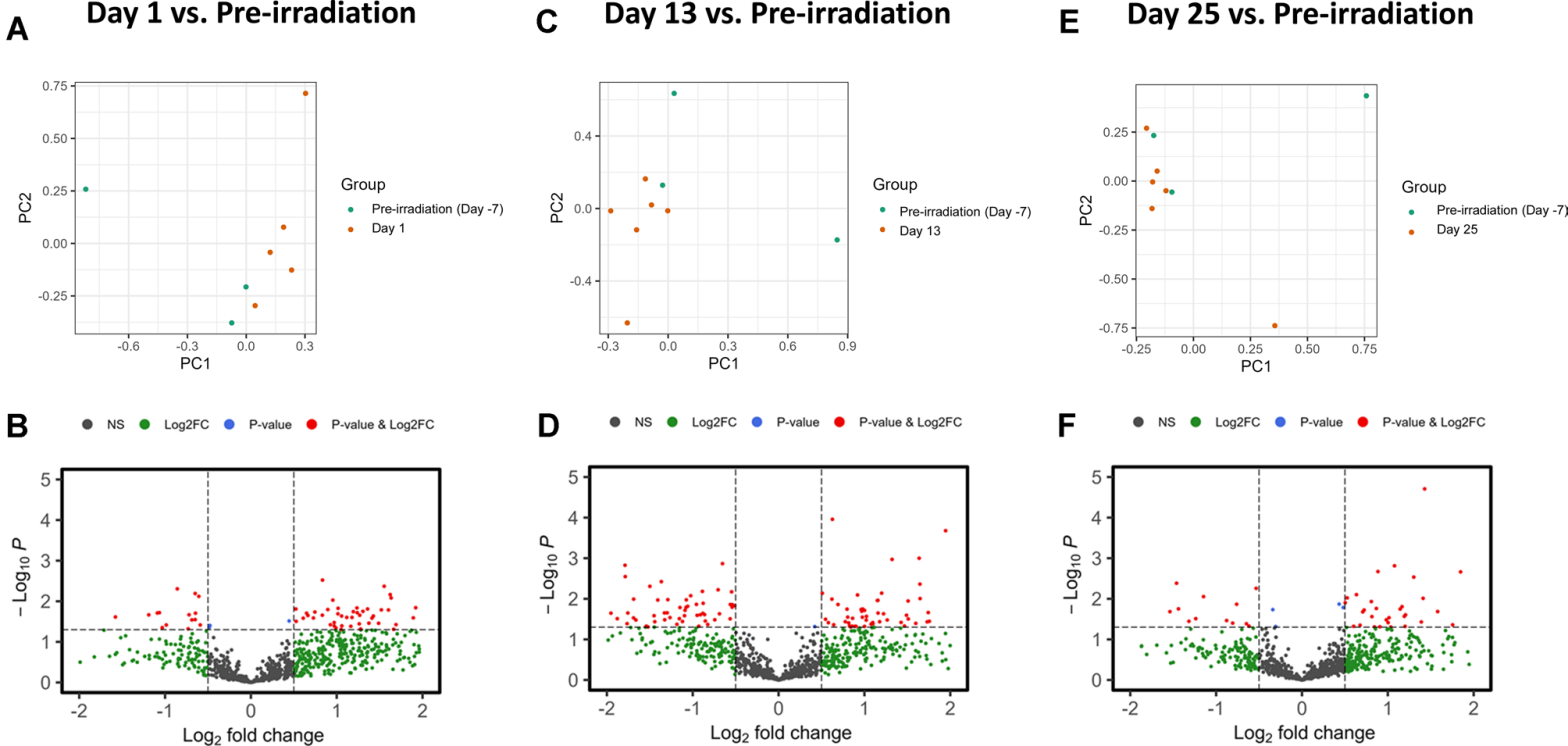
PCA and volcano plots illustrating radiation induced proteomic alterations at day 1 (panels **A** and **B**), day 13 (panels **C** and **D**), and day 25 (panels **E** and **F**) compared to the pre-irradiation state.

The subtle yet definitive changes in proteomic profiles following exposure to ionizing radiation can be viewed in the PCA and volcano plots in Fig. 3. The changes in plasma proteomic profiles associated with ionizing radiation exposure comparing pre-irradiation to days 1, 13, and 25 post-irradiation (Fig. 3: panels A, C, and E, respectively) were subtle. For each time point post-exposure vs. pre-exposure, PCA scores separated mostly along component one. This component one separation seemed to diminish as time post-exposure increased from day 1 to day 25. While there is some overlap in the PCA plots, suggesting there are some shared proteomic features, there are also distinct regions where the post-irradiation samples cluster away from the pre-irradiation group, indicating specific proteomic changes induced by radiation. The corresponding volcano plots (Fig. 3: panels B, D, and F) display metabolites that meet significance based on *p*-value (X-axis) and fold change (Y-axis), and reveal a more granular perspective. The majority of proteins do not display drastic changes in expression; however, there were a few select proteins that crossed the threshold of statistical significance and fold change.

**Figure 4:**
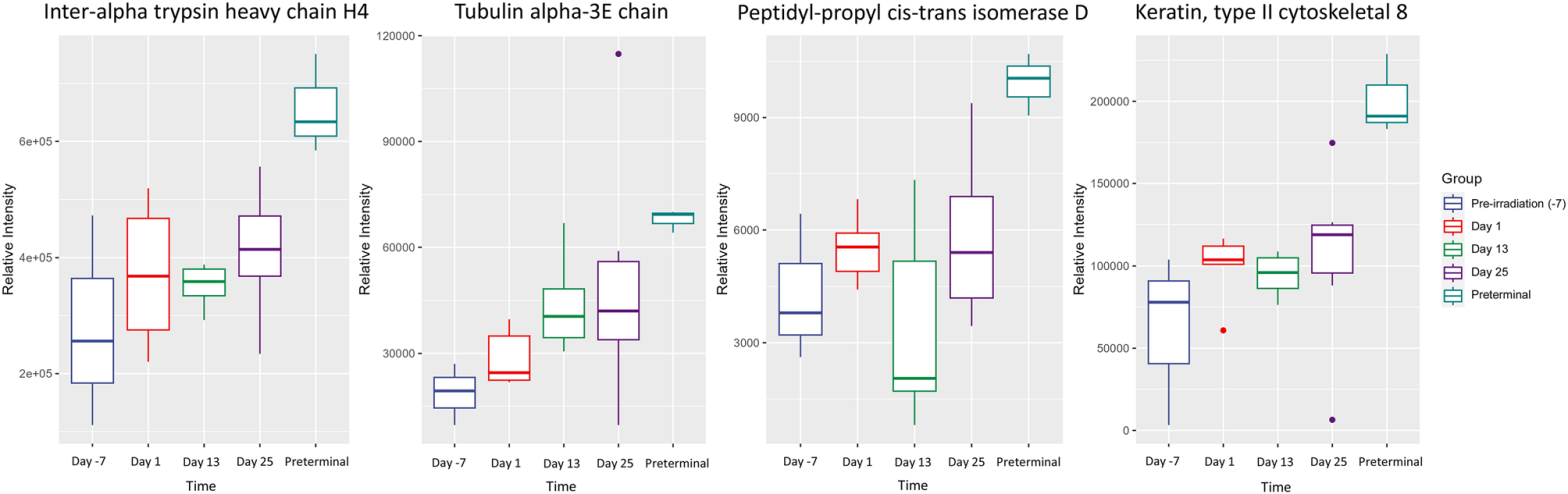
Boxplot graphs illustrating relative intensities of inter-alpha-trypsin inhibitor heavy chain H4, tubulin alpha 3U chain, peptidyl-prolyl cis–trans isomerase D, and keratin type II cytoskeletal 8 across pre-irradiation (day -7), day 1, day 13, day 25, and the preterminal group. Significant upregulation of these proteins was observed when comparing each post-irradiation time point to the preterminal group (the preterminal vs. day 25 comparison was not significant for tubulin alpha-3E chain).

Significant changes (FDR correction applied) in proteomic profiles were highest at days 1 and 13 post-irradiation (103 and 128 significantly dysregulated proteins, respectively) when comparing to the pre-irradiation time point. By day 25, many of these aberrations resolved, with only 62 dysregulated proteins remaining in surviving animals. Variability in protein expression was observed across all post-irradiation study days analyzed (days 1, 13, and 25). While there is a discernible overlap in the early post-irradiation stages, the distinction becomes more pronounced by the last study day (day 25) in several proteins. For example, there was an upregulation of inter-alpha trypsin inhibitor heavy chain H4, tubulin alpha-3E chain, peptidyl-prolyl cis–trans isomerase D, and keratin type II cytoskeletal 8 in response to radiation exposure, which continued to gradually increase as the study progressed suggesting a correlation with the preterminal phenotype (Fig. 4). Other proteins were significantly upregulated at all time points post-irradiation, which included UDP-GlcNAc:betaGal beta-1,3-N-acetylglucosaminyltransferase 7; lipopolysaccharide-binding protein; keratin, type II cytoskeletal 1b; Fer-1-like protein 4; dynein heavy chain domain-containing protein 1; dihydrolipoyl dehydrogenase, mitochondrial; complement C5; ceruloplasmin; and actin, alpha skeletal muscle (Table 1).

**Table 1:** Proteins that were significantly upregulated at all time points post-irradiation when comparing to the pre-irradiation baseline.

		Day 1 vs Pre-irradiation		Day 13 vs Pre-irradiation		Day 25 vs Pre-irradiation	
UniprotID	Protein name	Fold change	log2 (FC)	Fold change	log2 (FC)	Fold change	log2 (FC)
Q8NFL0	UDP-GlcNAc:betaGal beta-1,3-N-acetylglucosaminyltransferase 7	5.52	2.46	5.75	2.52	2.47	1.30
P18428	Lipopolysaccharide-binding protein	6.42	2.68	10.79	3.43	6.94	2.79
Q7Z794	Keratin, type II cytoskeletal 1b	2.66	1.41	3.85	1.95	3.42	1.77
A9Z1Z3	Fer-1-like protein 4	2.87	1.52	2.85	1.51	2.21	1.14
Q96M86	Dynein heavy chain domain-containing protein 1	1.68	0.75	1.79	0.84	1.81	0.86
P09622	Dihydrolipoyl dehydrogenase, mitochondrial	3.78	1.92	2.50	1.32	2.69	1.43
P01031	Complement C5	1.57	0.65	1.98	0.99	1.85	0.89
P00450	Ceruloplasmin	2.11	1.08	2.23	1.16	2.28	1.19
P68133	Actin, alpha skeletal muscle	6.18	2.63	5.45	2.45	3.60	1.85

### Marked yet variable responses in protein expression were noted in the preterminal state

**Figure 5:**
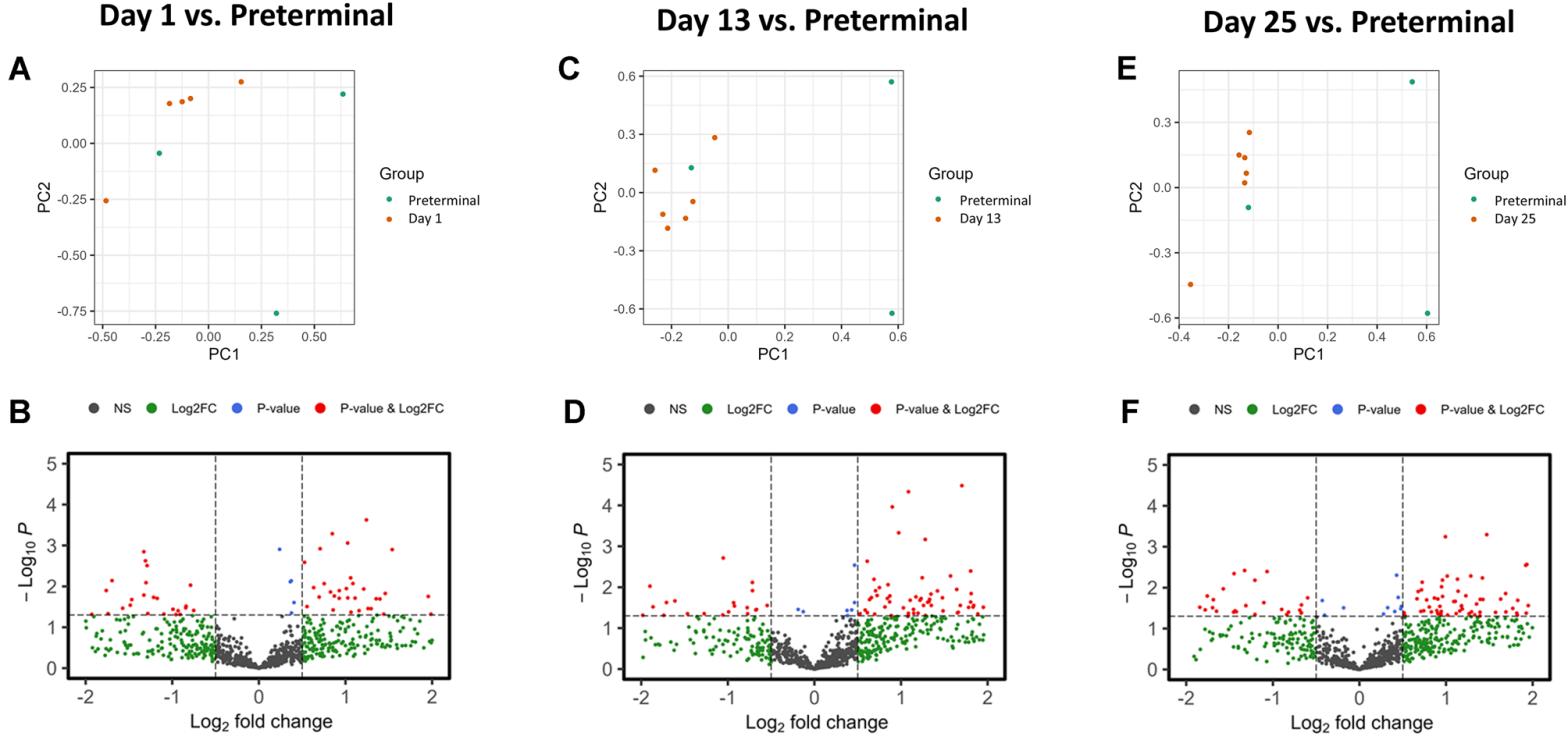
PCA and volcano plots illustrating radiation induced proteomic dysregulations at day 1 (Panels **A** and **B**), day 13 (Panels **C** and **D**), and day 25 (Panels **E** and **F**) compared to the preterminal state.

Pronounced and complex proteomic changes were noted as NHPs advanced to the preterminal phase. The PCA visualizations across comparisons with the day 1, day 13, and day 25 time points reveal a clear divergence in proteomic signatures in the preterminal group (Fig. 5: panels A, C, and E). The volcano plots reflect a marked increase in proteins crossing the threshold of significance within the preterminal group, indicating a heightened level of proteomic disruption as the animals approached terminal conditions (Fig. 5: panels B, D, and F). However, the spread of the data points suggests a varied response among proteins with considerable individual variation between animals.

**Figure 6:**
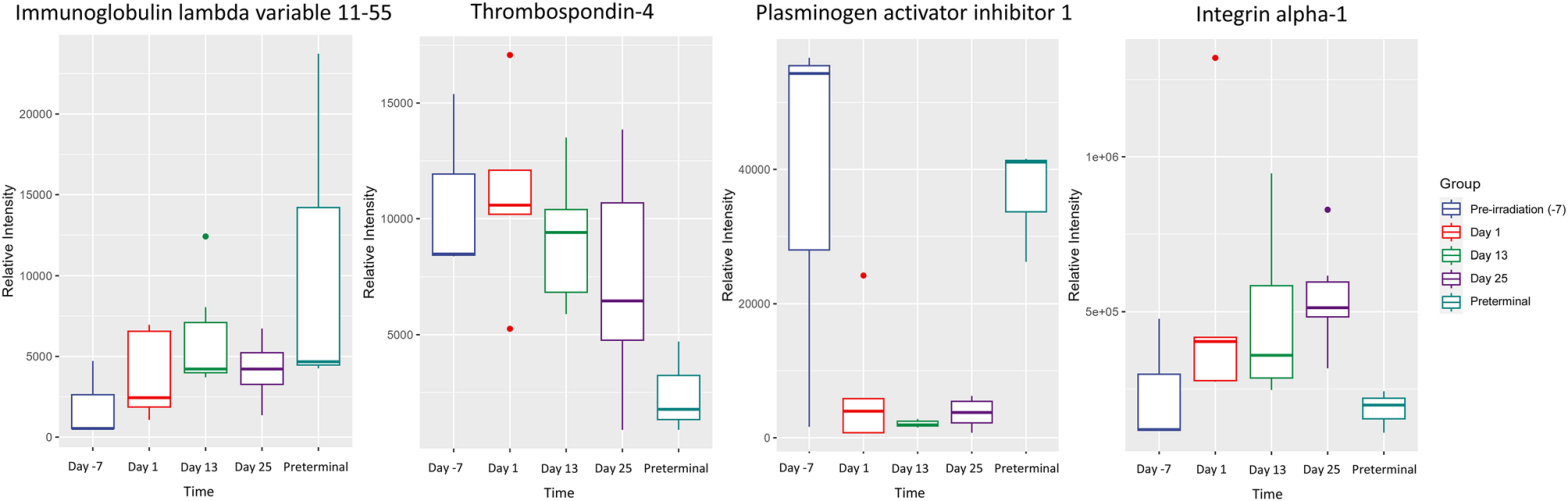
Boxplot graphs illustrating the relative intensities of immunoglobulin lambda variable 5–48, thrombospondin-4, plasminogen activator inhibitor 1, and integrin alpha-1 across pre-irradiation (day -7), day 1, day 13, day 25, and the preterminal group. Thrombospondin-4 and integrin alpha-1 were significantly downregulated in post-irradiation time points when compared to the preterminal group (apart from the day 25 comparison for thrombospondin-4), while plasminogen activator inhibitor 1 was significantly upregulated. Immunoglobulin lambda variable 11–55 was also upregulated in all post-irradiation time points, but none of these comparisons reached the threshold of significance.

As expected, a lesser degree of significance was noted when comparing preterminal samples to the post-irradiation time points, and these significant differences were more pronounced in the later study days (days 13 and 25). Inter-alpha-trypsin inhibitor heavy chain H4, tubulin alpha-3E chain, peptidyl-prolyl cis–trans isomerase D, and keratin type II cytoskeletal 8 expression increased gradually post-irradiation (apart from a decrease in intensity in peptidyl-prolyl cis–trans isomerase D on day 13), with a more marked and distinct increase as animals approached the preterminal state (Fig. 4). Other proteins followed more unique trends in preterminal statuses that varied in response, underscoring the complex and varied response to radiation in proteomic profiles (Fig. 6). Thrombospondin-4 and integrin alpha-1 were significantly downregulated when comparing the preterminal samples to post-irradiation samples, while plasminogen activator 1, inter-alpha-trypsin inhibitor heavy chain H4, keratin (type II cytoskeletal 8), peptidyl-prolyl cis–trans isomerase D, and tubulin alpha-3E chain were significantly upregulated. Uniquely, unlike the other significant proteins, integrin alpha 1 and Isoform 2 of Hyaluronidase-1 (Hyal-1) (EC 3.2.1.35) (Hyaluronoglucosaminidase-1) (Lung carcinoma protein 1) (LuCa-1) were significantly upregulated when comparing the pre-irradiation to post-irradiation time points, but significantly downregulated when comparing the preterminal samples to pre-irradiation samples. Conversely, plasminogen activator inhibitor 1 followed an opposite trend, and was downregulated in pre-irradiation vs. post-irradiation comparisons, but significantly upregulated in post-irradiation vs. preterminal comparisons. Immunoglobulin lambda variable 11–55 displayed a unique trend in intensity across the course of the study, which seemed to gradually increase and then decrease by day 25, with an increased expression in the preterminal state; this protein was upregulated in the preterminal vs. post-irradiation comparisons, but not significantly so.

## Discussion

The identification and validation of proteomic biomarkers for detection and/or prediction of radiation injury currently represents an unmet medical need. Interrogating longitudinally collected biospecimens pre- and post-irradiation for downstream molecular phenotyping analyses allows for the identification of several potential proteomic biomarkers. These biomarkers are indicators of overall health or decline thereof, and can be leveraged for early interventions and/or to manage ARS in exposed populations. Additionally, once validated, these biomarkers also have tremendous translational ability and many applications including drug development, understanding the effects of radiation on biological systems, and assessing absorbed radiation doses in exposed populations after a nuclear event.

Extensive research evaluating the changes within proteomic profiles incited by lethal doses of ionizing radiation has been conducted in our laboratory^23^. Serum samples of irradiated NHPs^24,25^, tissue (jejunum) and biofluids (serum) of irradiated mice^26,27^, in addition to irradiated CD^34+^ cell culture supernatants^28^ have been thoroughly evaluated. The radiation sources utilized in our studies contain high level cobalt-60 gamma radiation and various radiation countermeasures under development have been tested including tocopherol succinate^27^, gamma-tocotrienol^26,27^, BIO 300^24^, and Ex-Rad^25^. Tocopherol succinate and gamma-tocotrienol have been evaluated in murine models^26,27^ (tocopherol succinate was also investigated using CD^34+^ cells in vitro^28^), while BIO 300 and Ex-Rad have been investigated using NHP models^24,25^. To assess these proteomic changes, methods including NanoUPLC-MS/MS^24,25^, two-dimensional differential in-gel electrophoresis (2D-DIGE)^26,27^, and a high throughput antibody microarray platform^28^ have been used.

In this study, we aimed to characterize the proteomic changes induced by 7.2 Gy TBI by comparing samples collected before irradiation to samples collected post-irradiation at pre-selected time points (days 1, 13, and 25 post-irradiation). Plasma samples were also collected from moribund animals immediately prior to humane euthanasia; in this study, we have termed these samples “preterminal.” These preterminal samples were compared to the pre-irradiation and post-irradiation time points, and offer insight into the complex changes that are occurring on a cellular level in animals that are experiencing significant health decline and are on the verge of death.

As expected, a magnitude of overall difference was noted when comparing preterminal samples to the post-irradiation time points, and these significant differences were more pronounced in the later study days (days 13 and 25). Ultimately, although there was a clear delineation between the pre-irradiation and immediate post-irradiation (day 1) groups, the subsequent time points (day 13 and day 25) demonstrated a trajectory of proteomic alterations that trended toward downregulation, possibly reflecting a biological adaptation or progression of radiation-induced effects. In Fig. 3, which shows the pre-irradiation vs. post-irradiation comparisons, the PCA scores separations along component one suggest that radiation exposure produced a primary distinguishing response in the animals from which these samples were collected. That said, scores for samples post-irradiation did not cluster far from samples collected pre-exposure, a result when taken by itself indicates either considerable individual variation or a modest effect within these responses. Other evidence presented herein must be considered when exploring which conclusion is more accurate.

A deeper analysis revealed that radiation induced significant changes in inflammatory, hemostatic, and cellular structural proteins, suggesting these classes of proteins are detrimentally affected by radiation exposure, confirming previous research in which these radiation-induced changes are well-documented^21,25,29^. Radiation induces acute damage in both immune and hematopoietic cells, contributing to the development of ARS. However, the long-term immunological effects of radiation on the immune and hematopoietic systems are lesser known^30,31^. Additionally, it has been established that radiation has detrimental effects on the cell membrane, and this damage in turn initiates cellular apoptosis via signaling events^32^. However, heterogeneity in protein responses underscores the complexity of the NHP plasma proteome's reaction to radiation and the influence of individual physiological variability. In other words, a few proteins displayed consistent patterns in intensities post-irradiation, while others followed more unique trends in irradiated animals. Inter-alpha-trypsin inhibitor heavy chain H4, for example, plays an important role in inflammatory responses^33,34^. The trajectory of expression in this protein showed a strong positive correlation with proteomic changes in the preterminal phase in a time dependent manner, suggesting heightened biological stress or damage responses. Integrin alpha-1 and Isoform 2 of Hyaluronidase-1 (Hyal-1) (EC 3.2.1.35) (Hyaluronoglucosaminidase-1) (Lung carcinoma protein 1) (LuCa-1) followed unique trends among the proteins analyzed and were significantly upregulated in pre-irradiation vs. post-irradiation comparisons but significantly downregulated in preterminal vs. post-irradiation comparisons. These proteins play significant roles in regulating inflammation, tissue injury, and repair^35,36^. Thrombospondin-4 has been found altered in S-adenosylhomocysteine hydrolase deficiency^37^, atherosclerotic disease^38^, systemic lupus erythematosus^39^, gestational diabetes mellitus^40^, and several other states or conditions. Due to these differences in preterminal expression compared to post-irradiation expression, these proteins should be further investigated in future proteomic studies as possible indicators of health decline.

The effect of radiation on protein expression varied greatly in terms of patterns in up and downregulation, which further underscores the complex and varied response to radiation and suggests a cascade of biological events leading to a unique proteomic signature associated with the preterminal state. This disparity not only confirms the immediate effects of radiation but also indicates a progressive and compounded proteomic alteration over time, culminating in a distinct preterminal proteomic signature. These insights provide a valuable framework for understanding the progression of radiation effects on a proteomic level and aid in identifying potential biomarkers that could signal the beginning of the transition to critical health stages in irradiated organisms. This study is among the first proof of concept studies for delineating changes in protein expression that correlate with the preterminal stage. Previous transcriptomic and metabolomic research has determined that there are definitive proteomic signatures in preterminal statuses, which warrant additional investigations^21,22^.

In a previously performed metabolomics study, a distinguishable preterminal phenotype was observed in animals exposed to 7.2 Gy total-body radiation, with notable dysregulation in metabolites related to the glycerophospholipid metabolism and steroid hormone biosynthesis and metabolism pathways^21^. Notably, metabolomic and proteomic preterminal signatures were demonstrated in both this previous study and this current study. Although our results provide a strong proof of concept for delineation of protein biomarkers of the preterminal state, ultimately, continued research into the preterminal state of moribund NHPs is needed to further identify and validate proteins and pathways that can be targeted for the development of various therapeutic strategies to treat ARS. Further research is also needed to determine the mechanism underlying dysregulation of proteomic expression in response to radiation exposure; however, we posit that this is due at least in part to radiation-induced structural and functional damage to proteins leading to pathway perturbations, or perhaps even compensatory reactions to repair the radiation-induced damage. To this end, an ongoing study in our laboratory using similar preterminal samples from a large number of NHPs irradiated with two separate doses of cobalt-60 gamma-radiation will allow for the validation of this study’s results.

## Materials and methods

### Experimental design

The primary objective of this proteomic investigation was to discern changes in NHP plasma profiles in samples collected pre- and post-exposure to 7.2 Gy total-body gamma-radiation. Preterminal samples were also collected from moribund NHPs immediately prior to euthanasia and were compared to the pre-irradiation and post-irradiation time points. The experimental design of this study is presented in Fig. 1.

### Animals

 The samples of a total of 14 male NHPs (*Macaca mulatta,* age 3.0–5.3 years and weight 3.89–6.34 kg) used in a prior study were investigated in this study. Fourteen animals used in different prior experiments were included in this study. These animals were selected based on the availability of plasma samples. Six animal samples per group for each timepoint were compared. Only four preterminal plasma samples were collected immediately prior to euthanasia in moribund animals. The number of samples in the preterminal group were limited, either due to animal survival or inability to collect samples, which reflects the smaller number of available samples for comparison in this sample group (n = 4). All 14 animals were procured from the National Institutes of Health Animal Center located in Poolesville, MD. These NHPs were housed in a facility accredited by the Association for Assessment and Accreditation of Laboratory Animal Care (AAALAC)-International and underwent quarantine for seven weeks. Details of animal care are described earlier^21^. The study design and animal procedures were approved by the Institutional Animal Care and Use Committee of the Armed Forces Radiobiology Research Institute and the Department of Defense Animal Care and Use Review Office (ACURO). All animal procedures strictly adhered to the *Guide for the Care and Use of Laboratory Animals* throughout this study as described earlier^41,42^. This study was carried out in compliance with the ARRIVE guidelines.

### Irradiation

Animals were organized into groups for radiation exposure. The groups were then paired based on the similarities of their abdominal lateral separation measurements (+ /− 1 cm). These measurements were precisely obtained utilizing a digital caliper at the core of the abdomen. Animals whose abdomens were not measured within 1 cm of another animal's measurements were irradiated individually.

All NHPs underwent a fasting period 18 h prior to radiation exposure, in order to mitigate the risk of irradiation-induced vomiting. Animals were then sedated 15 min prior with 10–15 mg/kg of ketamine hydrochloride (100 mg/ml) injected intramuscularly (*im*). Thereafter, animals were placed in custom-made Plexiglas restraint boxes and secured. If needed, NHPs were administered a booster (0.1–0.3 ml *im*) of Ketamine hydrochloride prior to irradiation to reduce potential movement. Positioned in opposite directions on the irradiation platform, two NHPs were exposed to cobalt-60 total-body gamma radiation simultaneously at a dose of 7.2 Gy (dose rate of 0.6 Gy/min)^43^. When any two animals’ abdominal lateral separation measurements were not within + /− 1 cm, the two animals were irradiated individually.

Animals were irradiated between 8:00 AM and 12:00 PM. Following irradiation, animals were returned to their home cages and closely monitored until recovering from sedation. Additional details of TBI are given in earlier publications^44,45^. For dosimetry, the alanine/electron paramagnetic resonance (EPR) system was employed and is recognized as the most precise and accurate methods for measuring high radiation doses^46–48^.

### Cage-side animal observations

During the quarantine and study periods, cage-side observations of animals were preformed twice daily, once in the morning and once in the afternoon. Between days 10–20 post-irradiation, animals were observed three times a day approximately 6–8 h apart. Animals that met the criteria for euthanasia outlined in the study protocol were euthanized under the attending veterinarian’s suggestion. Several parameters were used as guidelines for moribundity including inappetence, severe anemia, weakness, minimal or no response to stimuli, etc.^42^.

### Blood sample collection

Blood was collected through a peripheral vessel (via the saphenous or cephalic vein) on days -7, 1, 13, and 25, as well as immediately prior to the euthanasia (preterminal) of moribund animals, as previously discussed^49^. Out of 14 animals, only four animals became moribund from which we could successfully collect preterminal samples. The remaining animals either survived or collection of samples prior to euthanasia was not possible. A 3 ml disposable luer-lock syringe with a 25-gauge needle was used to collect one ml of blood in an ethylenediaminetetraacetic acid (EDTA) tube. Samples were then centrifuged, and plasma was collected.

### Euthanasia

Although the selected study period was scheduled for 60 days, a couple of animals became moribund during the course of the study as a result of the LD_70/60_ radiation dose that was used (7.2 Gy total-body exposure). Euthanasia of the moribund animals was performed by a board-certified veterinarian or trained study staff in order to minimize pain and suffering. Animals were euthanized following the American Veterinary Medical Association (AVMA) guidelines^46,50^. To prepare for euthanasia, animals were sedated with Ketamine hydrochloride (5–15 mg/kg, *im*) injection. Euthanasia was performed by sodium pentobarbital administered intravenously (> 100 mg/kg, Euthasol, Virbac AH, Inc, Fort Worth, TX). Death was confirmed by cessation of pulse, heartbeat, and breathing.

### Plasma sample preparation

The Enrich iST 96X sample kit was used to produce the sample in accordance with the PreOmics manufacturer’s instructions. To summarize, 25 µL of EN-Beads were rinsed three times, and 20 µL of plasma was combined with 80 µL of EN-BIND buffer inside the EN-beads. The mixture was then incubated for 30 min at 30 °C and 1200 rpm. Following the three washing stages with the magnetic plate, 50 µL of LYSE-BCT was added to each bead pellet. The beads were then heated to 95 °C for 10 min while being shaken at 1000 rpm to reduce disulfide bridges, alkylate cysteines, and denature proteins.

Following a 5-min room temperature cooling phase, the mixture was supplemented with Trypsin and LysC, and the proteins were digested for one hour at 37 °C. The “Stop” solution was added to halt digestion, and three rounds of washing and elution into the collection plate using the supplied solutions followed to achieve peptide purification. Centrifugation was carried out for three minutes at 2250 g. According to the manufacturer's recommendations (ThermoFisher), peptides were measured using the Quantitative Fluorometric Peptide Assay, transferred to low-bind tubes, dried in a vacuum centrifuge, and then an estimated 500 ng of peptide per sample was resuspended in water with 0.1% FA for MS analysis.

### High-pH reverse-phase fractionation for library generation

To generate plasma proteome libraries, pools for each plasma sample were generated and pool plasma prepared according to the procedure above. The peptides were fractionated using the Pierce™ High pH Reversed-Phase Peptide Fractionation Kit into 10 fractions as described previously to generate deep proteomes. Peptides quantified using Quantitative Fluorometric Peptide Assay according to manufacture instructions (ThermoFisher) were transferred to low bind tubes, dried in a vacuum centrifuge, and an estimate of 500 ng of peptide per fractions was mixed resuspended in water with 0.1% FA for MS analysis.

### LC–MS/MS in DDA-PASEF and diaPASEF modes

Peptides from the individual fractions were separated by using a nanoElute 2 (Bruker Daltonik Scientific) coupled on-line to a timsTOF HT mass spectrometer (Bruker Daltonik). Peptides were analytically separated on a PepSep25 column (75 μm × 25 cm, 1.5 μm, C18) and heated to 50 °C at a flow rate of 400 nl/min. LC mobile phases A and B were water with 0.1% FA (v/v) and ACN with 0.1% FA (v/v), respectively. The nanoLC was coupled to the timsTOF Pro via a modified nanoelectrospray ion source (Captive Spray II; Bruker Daltonik). Initially, 90 min gradient for the fractionated peptides from QC samples were separated. Data acquisition on the timsTOF HT was performed using TIMSControl 6.0 (Bruker Daltonik) in DDA_PASEF method with the following parameter: accumulation and ramp time were set to 100 ms each. Mass spectra were recorded in the range from m/z 100 to 1700. The ion mobility was scanned from 0.85 to 1.35 (V·s)/cm^2^. Precursors for data-dependent acquisition were isolated within ± 1 Th and fragmented with an ion mobility dependent collision energy, which was linearly increased from 20 to 59 eV. The overall acquisition cycle of 1.17 s comprised one full TIMS-MS scan and 10 parallel accumulation serial fragmentation (PASEF) MS/MS scans.

Proteomics data from each fraction samples were analyzed in Realtime PaSER software, searched against the human Swiss-Prot database with the species taxonomy set to *Homosapiens.* These files were used to generate spectral library for dia_PASEF method.

For diaPASEF acquisition, the capillary voltage was set to 1600 V. The MS1 and MS2 spectra were obtained over a mass-to-charge (m/z) range of 100–1700 Th, with an ion mobility range (1/K0) of 0.8–1.3 Vs/cm^2^. The other setting was the same as DDA-PASEF mode. Additionally, 28 Th width isolation windows were associated with ion mobility windows of 0.3 1/K0 to cover as close as possible the peptide-ions distribution on both m/z and mobility dimensions. Raw data of DIA were processed against the spectral library created from DDA-PASEF mode.

### General dia-PASEF analysis

Protein identification and quantification analysis were done with PaSER (2023, v 3.0, Bruker Scientific LLC, Billerica, MA, http://www.bruker.com) using TIMS DIA-NN. Mass spectra were streamed via the PaSER plugin directly from the timsTOF’s acquisition control software (timsControl) to the PaSER workstation via a dedicated LAN connection and pre-processed into a binary file for consumption by TIMS DIA-NN. A spectral library consisting of precursors including peptide modification such as phosphorylated and acetylated was re-annotated against Uniprot human protein database (downloaded on 01-01-2023) plus sequences of known contaminants such as keratin and porcine trypsin. 20 ppm precursor tolerance and 15 ppm fragment ion tolerance were used along with Top 3 precursors for quantitation. Multiple samples were assembled and match-between-runs was performed to fill-in missing values with an outlier frequency of 0.2, following which global normalization was performed.

### Statistical analysis

To evaluate the effects of 7.2 Gy TBI on NHP plasma profiles, protein abundance, represented by normalized intensity units, was compared. A comprehensive list of all proteins screened for in this study can be viewed in Supplementary Table 1. The blood plasma profiles collected at various timepoints (day 1, day 13, or day 25) were compared to samples collected pre-irradiation and immediately prior to euthanasia (preterminal). Both independent (unpaired) and dependent (paired) non-parametric statistical tests were performed, and these results can be viewed in Supplementary Tables 2 and 3, respectively. Additionally, Supplementary Table 4 combines all comparisons for a holistic view across all groups and time points. For nonparametric data analysis, Mann–Whitney U tests were performed for unpaired comparisons, while for paired comparisons, the Wilcoxon signed-rank test was utilized. A *p*-value of less than 0.05 was considered statistically significant. Additionally, in an effort to address the issue of multiple comparisons that can potentially increase the likelihood of false positives, the FDR method was applied to adjust *p*-values^51,52^. A more detailed summary of the statistical analyses used to analyze this data has been discussed in a recently published paper^21^.

### Ethical approval

The study was conducted in accordance with the Declaration of Helsinki, and approved by The Institutional Animal Care and Use Committee—Armed Forces Radiobiology Research Institute Approval Code: 2015-12-010, Approval Date: February 24, 2016. Department of Defense second tier approval: Department of Defense Animal Care and Use Review Office (ACURO) Approval Code: 2015-12-010, Approval Date: March 02, 2016.

### Supplementary Information

Supplementary Tables.

## Data Availability

All relevant data are within the manuscript and its Supporting Information files.
